# Adapting Montessori Programming for Aging and Dementia With Implementation Strategies

**DOI:** 10.1093/geroni/igab046.187

**Published:** 2021-12-17

**Authors:** Natalie Douglas

**Affiliations:** Central Michigan University, Mt Pleasant, Michigan, United States

## Abstract

There is a need to translate research findings to support the wider adoption of person-centered care into typical long-term care environments across the world. Montessori for Aging and Dementia is one mechanism to support person-centeredness, dignity and autonomy of older adults living in long-term care environments. In this presentation, strategies used to support the implementation of Montessori for Aging and Dementia in a long-term care community of 20 people living with severe dementia will be highlighted. Implementation support was provided through capturing and sharing local knowledge, ongoing training and consultation, and tailoring communication supports. Through the use of these iterative strategies, the program was successfully adapted to include people living with severe dementia. While key findings of the project included improvements on a variety of observational and staff administered measures, the focus of this presentation will be on the relationships between the Montessori program’s fidelity, local needs and implementation strategies.

